# THRSP identified as a potential hepatocellular carcinoma marker by integrated bioinformatics analysis and experimental validation

**DOI:** 10.18632/aging.203900

**Published:** 2022-02-23

**Authors:** Yuxi Ding, Xiaoling Liu, Yue Yuan, Yunjian Sheng, Decheng Li, Suvash Chandra Ojha, Changfeng Sun, Cunliang Deng

**Affiliations:** 1 The Department of Infectious Diseases, The Affiliated Hospital of Southwest Medical University, Luzhou 646000, China; 2 The Department of Tuberculosis, The Affiliated Hospital of Southwest Medical University, Luzhou 646000, China; 3 Laboratory of Infection and Immunity, The Affiliated Hospital of Southwest Medical University, Luzhou 646000, China

**Keywords:** THRSP, hepatocellular carcinoma, prognosis, invasion, migration

## Abstract

Hepatocellular carcinoma (HCC) is the most common malignant liver tumor with high mortality and poor prognosis worldwide. This study aimed to identify hub genes and investigate the underlying molecular mechanisms in HCC progression by integrated bioinformatics analysis and experimental validation. Based on the Gene Expression Omnibus (GEO) databases and The Cancer Genome Atlas (TCGA), 12 critical differential co-expression genes were identified between tumor and normal tissues. Via survival analysis, we found higher expression of LCAT, ACSM3, IGF1, SRD5A2, THRSP and ACADS was associated with better prognoses in HCC patients. Among which, THRSP was selected for the next investigations. We found that THRSP mRNA expression was negatively correlated with its methylation and closely associated with clinical characteristics in HCC patients. Moreover, THRSP expression had a negative correlation with the infiltration levels of several immune cells (e.g., B cells and CD4+ T cells). qRT-PCR verified that THRSP was lower expressed in HCC tissues and cell lines compared with control. Silencing of THRSP promoted the migration, invasion, proliferation, and inhibited cell apoptosis of HCCLM and Huh7 cell lines. Decreased expression of THRSP promoted HCC progression by NF-κB, ERK1/2, and p38 MAPK signaling pathways. In conclusion, THRSP might serve as a novel biomarker and therapeutic target of HCC.

## INTRODUCTION

Primary liver cancer is one of the most frequently diagnosed malignant tumors and the third leading cause of cancer-related mortality worldwide, with an estimated 906,000 new cases and 830,000 deaths in 2020 [1]. Hepatocellular carcinoma (HCC) is the most common form of primary liver cancer (accounting for 75–85%) [1]. Due to the lack of understanding about the complex carcinogenic mechanisms and efficient therapeutic targets, the 5-year survival rate for HCC patients remains poor [2]. Thus, there is an urgent need to seek promising targets and elaborate on the underlying molecular mechanisms involved in HCC progression.

The major public databases such as GEO (http://www.ncbi.nlm.nih.gov/geo/) and TCGA (https://portal.gdc.cancer.gov/), containing gene expression profiles, provide an opportunity to screen the differentially expressed genes (DEGs) related to the carcinogenesis and development of HCC [3, 4]. The cancer progression is regulated by the key modulators of gene-gene interaction networks, thus the Weighted Gene Co-expression Network Analysis (WGCNA) and protein-protein interaction (PPI) network analysis have been widely used to screen co-expressed genes that drive cancers [5, 6].

In this study, we performed differential gene expression analysis, WGCNA, and PPI network analysis to screen crucial differential co-expression genes associated with hepatocarcinogenesis based on the GEO and TCGA databases. Via 5-year survival analysis, we found that six genes (LCAT, ACSM3, IGF1, SRD5A2, THRSP, and ACADS) were associated with the prognosis of HCC patients. THRSP (thyroid hormone-responsive, also known as Spot 14 or S14) was originally identified in 1982 owing to its significant and rapid induction by thyroid hormone and it had been reported to have great effects on the tissue-specific regulation of lipid metabolism [7, 8]. Some studies demonstrated that THRSP was relatively abundant in liver, white and brown adipose, and lactating mammary tissues and it was associated with nonalcoholic fatty liver disease [9, 10]. THRSP was strongly expressed in most lipogenic breast cancers, and high expression of THRSP predicted a high recurrence rate of primary invasive breast cancers. THRSP mediated lipogenic effects of progestin, and THRSP knockdown disrupted lipid synthesis and induced apoptosis of breast cancer cells [11]. Another study reported that over-expression of THRSP increased medium-chain fatty acids synthesis and cell proliferation, but reduced tumor metastasis [12]. However, THRSP was found to be down-regulated in HCC tissues, and the decreased expression of THRSP was associated with worse prognosis in our study. To understand the roles of THRSP in HCC progression, we further analyzed the biological function and clinical implications of THRSP via integrated bioinformatics analysis. Moreover, we performed qPCR and immunohistochemical experiments to explore the mRNA and protein expression of THRSP in HCC tissues and cells. We also did Western blotting, CCK-8, Transwell, wound scratch and flow cytometry assays to investigate the function and molecular mechanism of THRSP in HCC.

## RESULTS

### Identification of crucial modules by WGCNA

**Figure 1:**
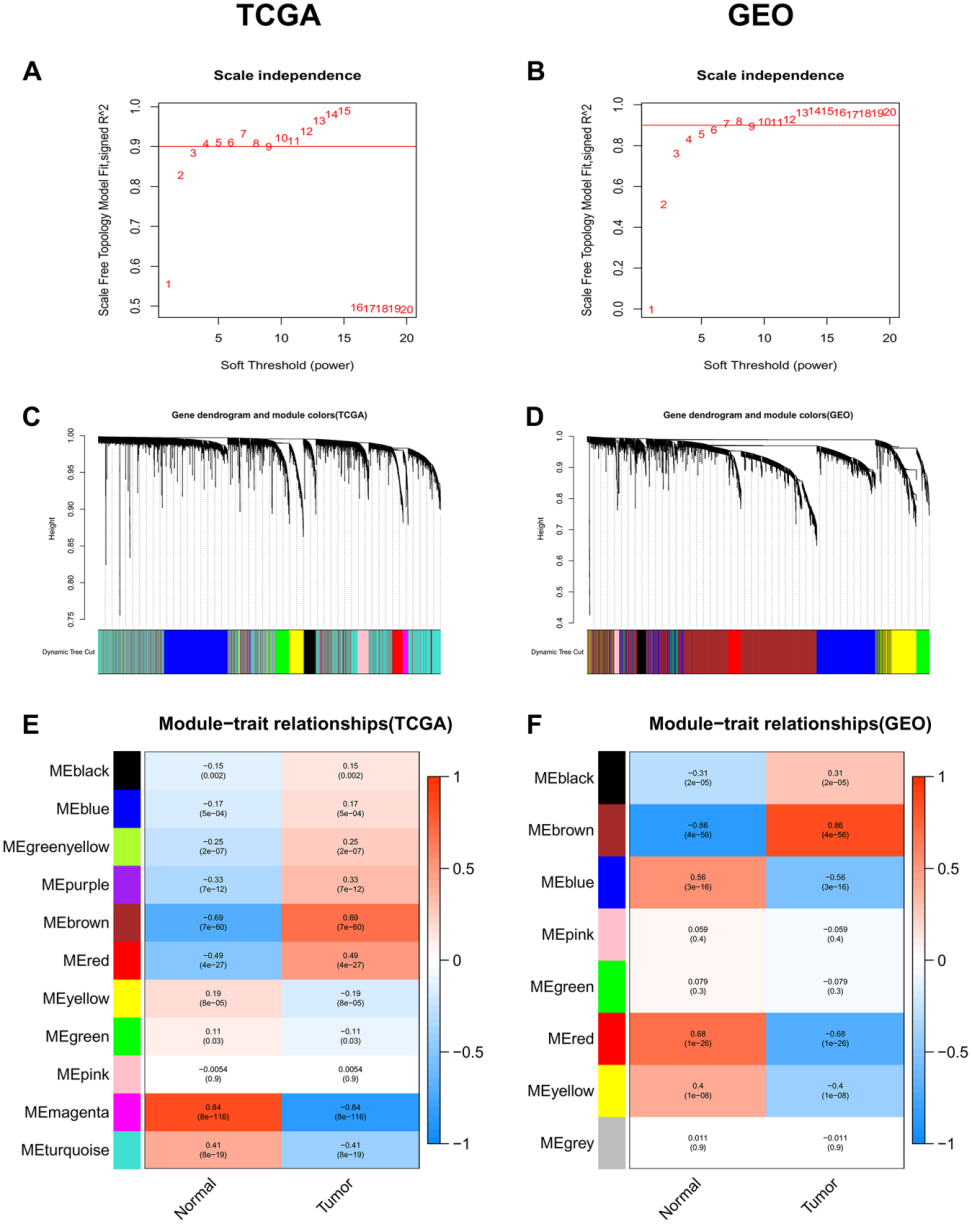
**Identification of co-expression modules associated with the clinical traits in the TCGA-LIHC dataset and two GEO datasets.** (**A**, **B**) Analysis of the scale-free fit index for various soft-thresholding powers (β). (**C**, **D**) Dendrogram of all genes in the TCGA-LIHC dataset or GEO datasets clustered based on the 1-TOM matrix. (**E**, **F**) Correlation between modules and clinical traits (normal and tumor). Each cell contains the corresponding correlation coefficient (the upper number) and the P-value (the lower number).

The “WGCNA” package was used to group genes into modules by the average linkage hierarchical clustering. In this study, the soft powers β = 3 and 5 were selected as the soft-thresholding to ensure scale-free networks (Figure 1A, 1B), and 11 modules in the TCGA-LIHC (Figure 1C) and 8 modules in the GEO datasets (Figure 1D) were generated. The heatmaps (Figure 1E, 1F) of module-trait relationships were plotted to identify modules most significantly correlated with clinical features (normal and tumor). We found the brown modules in the TCGA-LIHC (containing 2057 co-expression genes) and GEO datasets (containing 2145 co-expression genes) had the highest association with tumor tissues (brown module in TCGA-LIHC: r = 0.69, p = 7e-60; brown module in GEO datasets: r = 0.86, p = 4e-56), which were selected as modules of interest for the subsequent analysis.

### Identification of DEGs and differentially co-expressed genes

**Figure 2:**
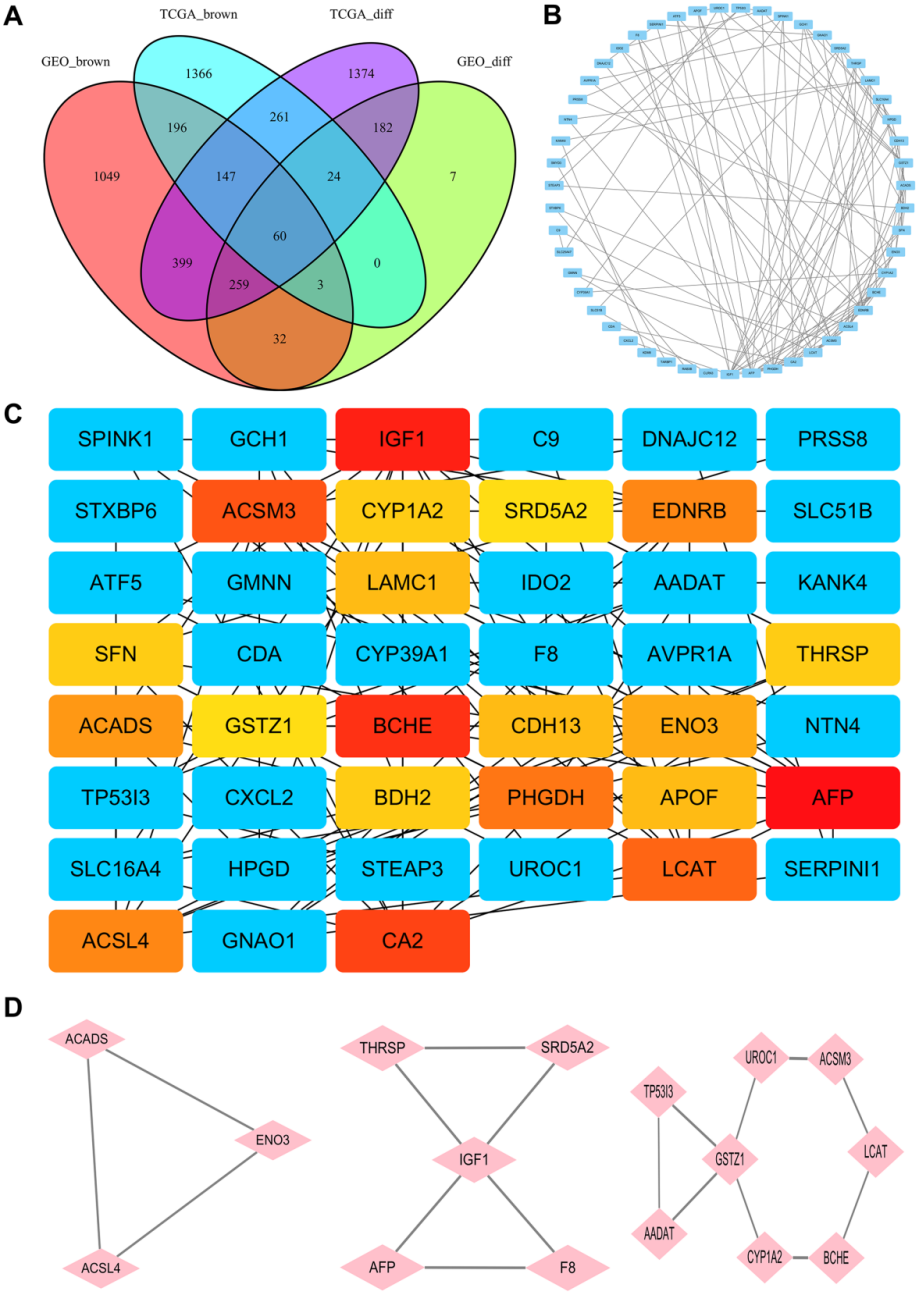
**Identification of hub genes.** (**A**) The Venn diagram for selection of the differential co-expression genes among DEG lists and co-expression modules. (**B**) PPI network of the intersection genes between DEG lists and co-expression modules. Each blue node represents a gene. Edges among nodes indicate interaction associations between genes. (**C**) Identification of the core genes from the PPI network by MCC algorithm. Darker colors refer to higher MCC sores. (**D**) The top three significant modules of the PPI network were evaluated in MOCDE. Pink nodes represent genes in corresponding modules.

After normalization of the microarrays, 2705 differentially expressed genes (DEGs) between the HCC and normal tissues from the TCGA dataset (TCGA_diff) and 567 DEGs from the GEO datasets (GEO_diff) were screened by the “limma” package in R. Then, a Venn diagram was performed to examine the intersection among the DEGs and co-expressed genes of key modules. As shown in Figure 2A, 60 differentially co-expressed genes were finally obtained.

### PPI network and hub genes

**Figure 3:**
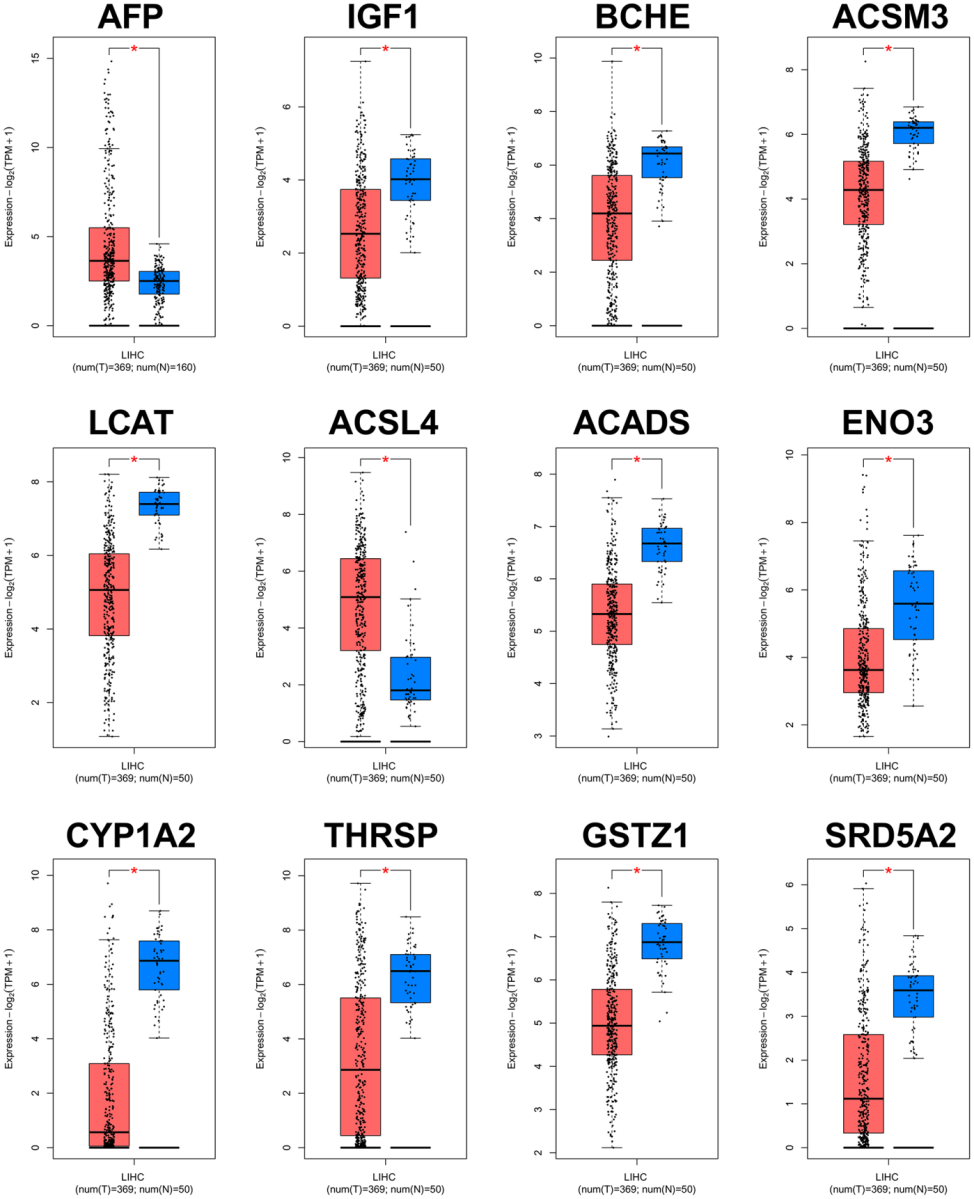
**Validation of expression levels of the 12 hub genes in HCC and normal tissues using GEPIA.** *P<0.01 is considered statistically significant. Tumor tissues are shown in red, and normal liver tissues are shown in blue.

**Figure 4:**
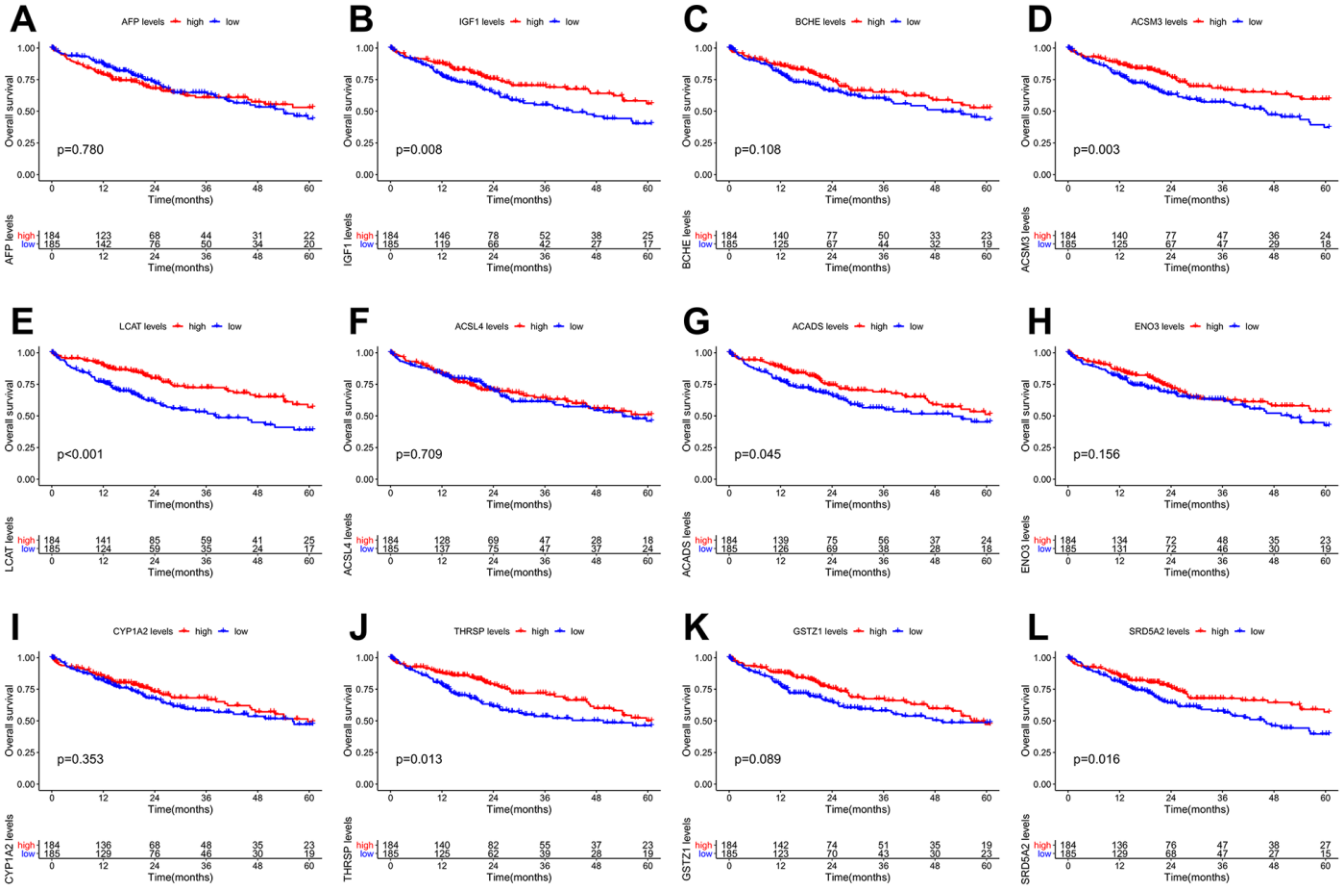
**Overall survival (OS) Kaplan-Meier curves of the 12 hub genes in HCC patients.** (**A**) AFP, p = 0.780. (**B**) IGF1, p = 0.008. (**C**) BCHE, p = 0.108. (**D**) ACSM3, p = 0.003. (**E**) LCAT, p <0.001. (**F**) ACSL4, p = 0.709. (**G**) ACADS, p = 0.045. (**H**) ENO3, p = 0.156. (**I**) CYP1A2, p = 0.353. (**J**) THRSP, p = 0.013. (**K**) GSTZ1, p = 0.089. (**L**) SRD5A2, p = 0.016.

The PPI network of the 60 differentially co-expressed genes was constructed in the STRING database (Figure 2B). As showed in Figure 2C, the core genes were extracted from the PPI network by the MCC algorithms via the CytoHubba plug-in. Meanwhile, the significant modules of the 60 differential co-expression genes were established using the MCODE plug-in in Cytoscape (Figure 2D). Combining the above two algorithms, AFP, IGF1, BCHE, ACSM3, LCAT, ACSL4, ACADS, ENO3, CYP1A2, THRSP, GSTZ1, and SRD5A2 were finally selected as hub genes. Among them, AFP and ACSL4 were up-regulated and the other 10 hub genes were down-regulated in HCC tissues (Figure 3). To evaluate the prognostic values of the hub genes in HCC patients, a 5-year survival analysis was performed by Kaplan-Meier plotter. As shown in Figure 4, higher expression of LCAT, ACSM3, IGF1, SRD5A2, THRSP, and ACADS was associated with a better 5-year overall survival (p<0.05).

### THRSP was down-regulated in HCC tissues and cell lines

**Figure 5:**
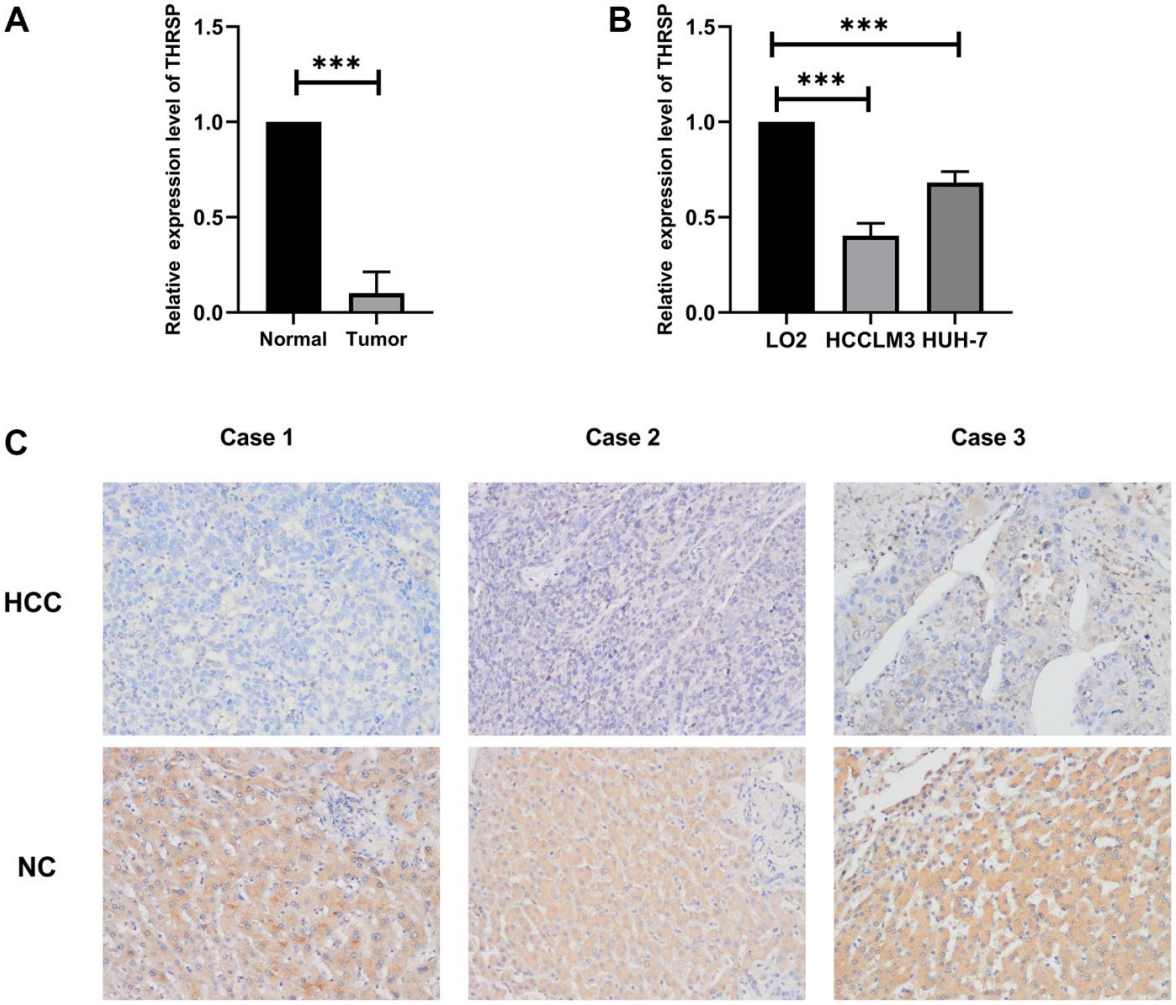
**The expression of THRSP at mRNA and protein levels.** (**A**, **B**) The mRNA expression of THRSP in HCC tissues and cells compared with control examined by RT-qPCR. (**C**) The protein expression of THRSP in HCC tissues and the adjacent normal tissues examined by immunohistochemical (200×).

To verify the expression of THRSP in HCC tissues and cell lines, the quantitative real-time PCR (qRT-PCR) and immunohistochemistry (IHC) assays were performed in this study. The results of qRT-PCR verified that THRSP mRNA expression was remarkably lower in HCC tissues as well as in HCCLM3 and Huh-7 cell lines compared with normal groups (Figure 5A, 5B). The results of IHC indicated that THRSP was down-regulated in most of HCC samples (8/10) (Figure 5C).

### The correlation between THRSP expression and clinical characteristics

Based on data from Illumina HumanMethylation 450 platform, we found a significant negative correlation (r = −0.81, p <0.001) between THRSP mRNA expression and methylation (Supplementary Figure 1A). Four THRSP promoter CpG sites were shown in Supplementary Figure 1B. The Spearman correlation analysis demonstrated that CpG sites were negatively correlated with the expression of THRSP (Supplementary Figure 1C–1F). Wilcox test was used to compare the difference in THRSP expression or methylation in groups divided by age, family history of cancer, gender, grade, Ishak fibrosis score and race. The details were shown in Supplementary Figures 2, 3. Then, the HCC patients were dichotomized into two groups (“low” or “high”) based on their THRSP expression levels or THRSP methylation levels using the median values as the cutoff point. The chi-square test was used to evaluate the correlation of THRSP expression or THRSP methylation with clinical characteristics. As listed in Table 1, THRSP mRNA expression or methylation was closely associated with the clinical indicators including age, family history of cancer, Ishak fibrosis score and gender et al.

**Table 1:** Correlations between THRSP expression/methylation and clinical features.

**Clinical features**		**THRSP expression**		**P value**	**THRSP methylation**		**P value**
		**Low (%)**	**High (%)**		**Low (%)**	**High (%)**	
Age	<=65	128(68.45)	107(57.53)	0.0378	108(57.75)	127(68.28)	0.0457
	>65	59(31.55)	79(42.47)		79(42.25)	59(31.72)	
Cancer Status	Tumor-free	79(55.24)	83(58.04)	0.7204	85(59.44)	77(53.85)	0.4036
	With tumor	64(44.76)	60(41.96)		58(40.56)	66(46.15)	
Grade	G1	16(8.65)	39(21.2)	6.00E-04	39(21.08)	16(8.7)	3.00E-04
	G2	86(46.49)	92(50)		94(50.81)	84(45.65)	
	G3	74(40)	50(27.17)		49(26.49)	75(40.76)	
	G4	9(4.86)	3(1.63)		3(1.62)	9(4.89)	
Stage	Stage I	78(44.57)	95(54.29)	0.1955	94(53.71)	79(45.14)	0.2653
	Stage II	48(27.43)	39(22.29)		41(23.43)	46(26.29)	
	Stage III	45(25.71)	40(22.86)		39(22.29)	46(26.29)	
	Stage IV	4(2.29)	1(0.57)		1(0.57)	4(2.29)	
Family history of	NO	114(70.81%)	96(59.63%)	0.0467	96(59.63)	114(70.81)	0.0467
cancer	YES	47(29.19%)	65(40.37%)		65(40.37)	47(29.19)	
Living status	Alive	117(62.57)	126(67.74)	0.3472	128(68.45)	115(61.83)	0.2175
	Dead	70(37.43)	60(32.26)		59(31.55)	71(38.17)	
Ishak fibrosis score	0	28(25.93)	47(43.93)	0.0421	48(44.44)	27(25.23)	0.058
	1,2	18(16.67)	13(12.15)		14(12.96)	17(15.89)	
	3,4	17(15.74)	11(10.28)		12(11.11)	16(14.95)	
	5,6	45(41.67%)	36(33.64%)		34(31.48%)	47(43.93%)	
Gender	Female	71(37.97)	50(26.74)	0.0271	48(25.67)	73(39.04)	0.008
	Male	116(62.03)	137(73.26)		139(74.33)	114(60.96)	
Race	Asian	96(53.04)	64(35.36)	9.00E-04	64(35.36)	96(53.04)	9.00E-04
	Black	4(2.21)	13(7.18)		13(7.18)	4(2.21)	
	White	81(44.75)	104(57.46)		104(57.46)	81(44.75)	
expression	Low	-	-	-	26(13.9%)	161(86.1%)	< 0.001
expression	High	-	-		161(86.1%)	26(13.9%)	
methylation	Low	26(13.9%)	161(86.1%)	< 0.001	-	-	-
methylation	High	161(86.1%)	26(13.9%)		-	-	

### Relationship of THRSP with immune infiltration based on TIMER 2.0

As illustrated in the scatter plots (Supplementary Figure 4), THRSP expression was negatively correlated with infiltrating levels of B cells, CD4+ T cells, dendritic cells, and positively correlated with CD8+ T cells, but was not correlated with macrophages or neutrophils. In addition, we investigated the correlations of THRSP expression with the gene markers of various immune immune-infiltrating cells (including T cell, B cell, monocyte, neutrophil, dendritic cell, tumor-associated macrophage and different types of functional T cells) in HCC with the TIMER 2.0 database. As listed in Supplementary Table 1, THRSP expression was negatively associated with most of the gene markers.

### Silencing of THRSP promoted HCC progression

**Figure 6:**
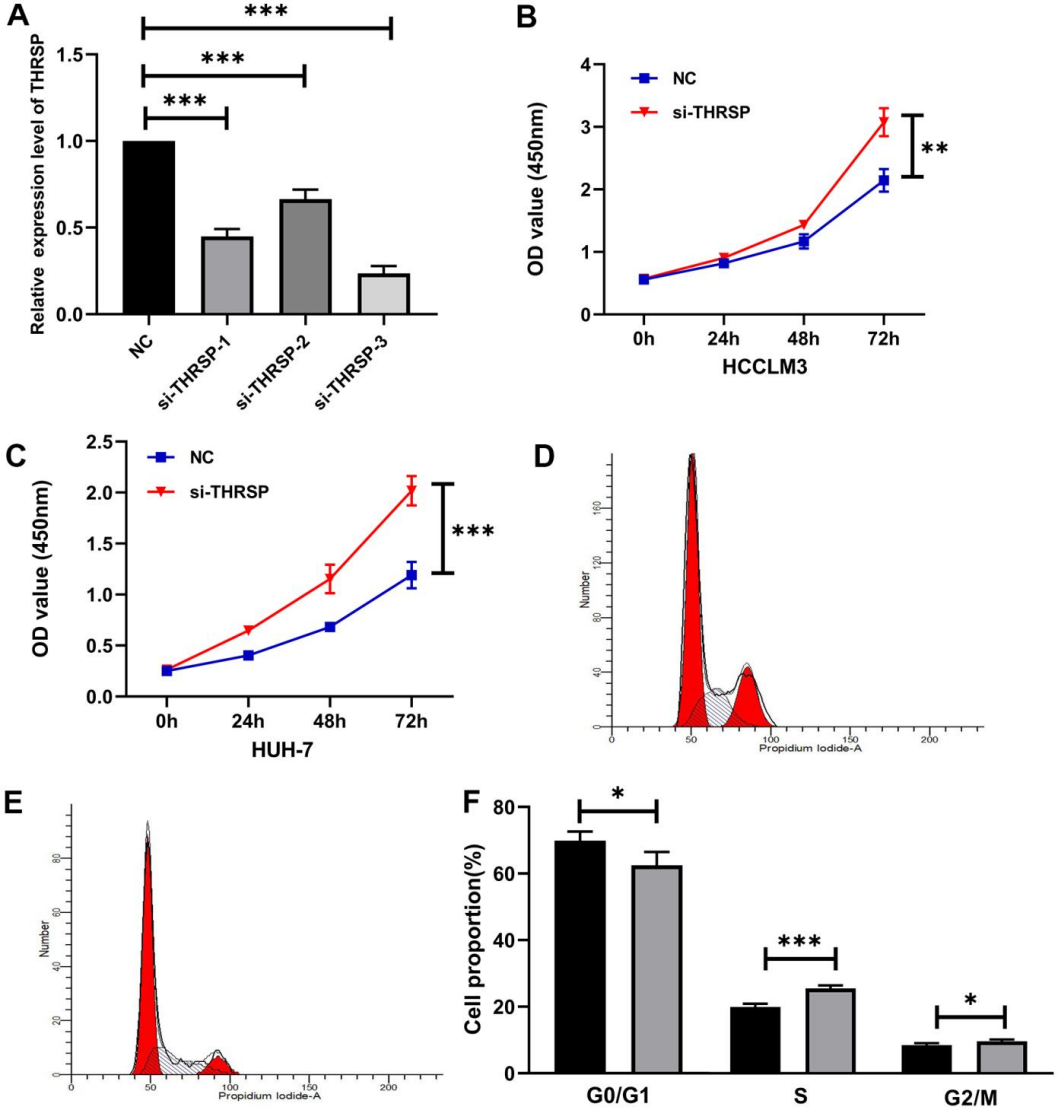
**The effect of THRSP expression on proliferation and cell cycle of HCC cells.** (**A**) The efficiency of the THRSP silencing determined by qRT-PCR. (**B**, **C**) The proliferation of HCCLM3 and Huh-7 cells examined by CCK-8. (**D**–**F**) The cell cycle assay detected by flow cytometry. NC group: black bars; si-THRSP group: grey bars. *p<0.05, **p<0.01, ***p<0.001.

**Figure 7:**
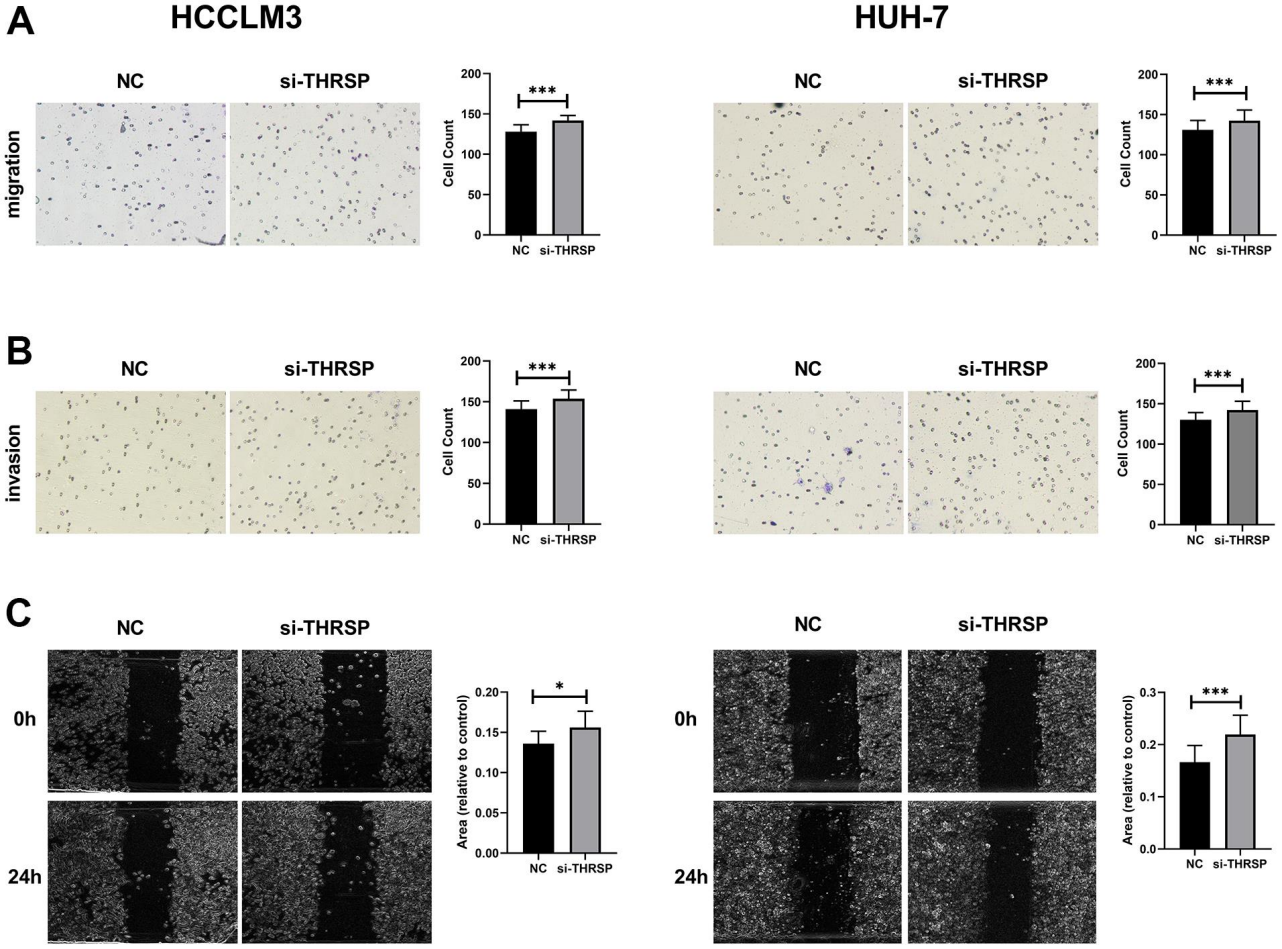
**Transwell and wound healing assays.** (**A**, **B**) The migration and invasion of HCCLM3 and Huh-7 cells detected by Transwell assays (magnification: 200×). (**C**) The migration ability of HCCLM3 and Huh-7 cells examined by wound healing assays (magnification: 40×).

To explore the functional roles of THRSP in HCC, three candidate siRNAs of THRSP (THRSP-siRNAs) were transfected into HCCLM3 and Huh-7 cells. qRT-PCR was performed to evaluate the inhibition efficiency of the three THRSP-siRNAs. Three THRSP-siRNAs significantly inhibited THRSP expression compared with the negative control (Figure 6A). The si-THRSP-3 was selected for the following experiments due to its robust silencing efficiency. The CCK-8 assays indicated that silencing of THRSP could promote proliferation of HCCLM3 and Huh7 cells (Figure 6B, 6C). The flow cytometry assays indicated that the si-THRSP group presented a significantly higher percentage of HCCLM3 cells in the S phase and the G2/M phase, but a lower percentage of cells in the G0/G1 phase compared with the control group (Figure 6D–6F). Transwell and wound scratch assays indicated that silencing of THRSP also promoted migration and invasion of HCC cells (Figure 7A–7C).

### Silencing of THRSP inhibited apoptosis of HCC cells

**Figure 8:**
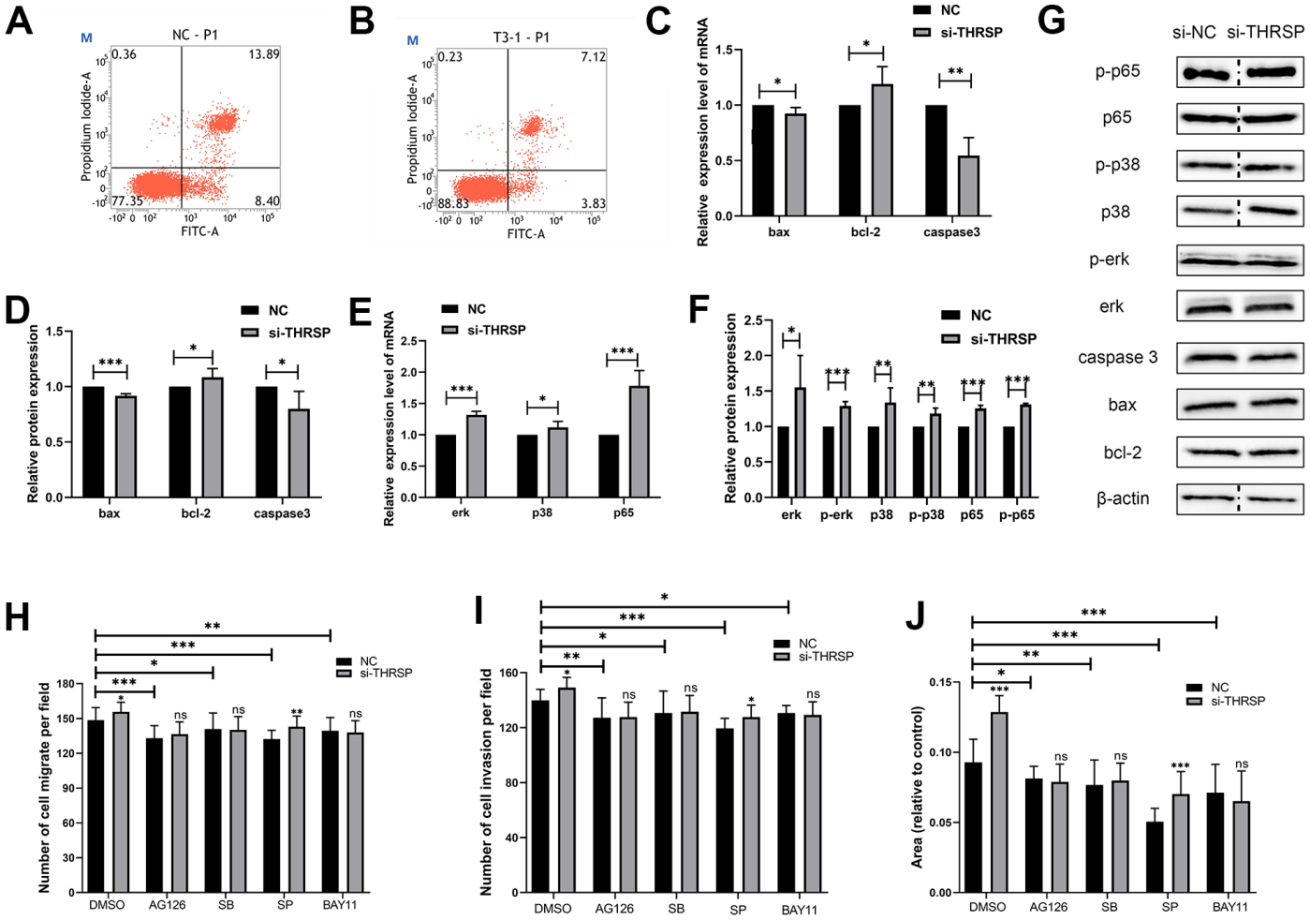
**The effect of THRSP expression on cell apoptosis and channel regulation in HCC.** (**A**, **B**) The apoptosis of HCCLM3 cells detected by flow cytometry. (**C**–**F**) The expression of apoptosis-related molecules (bax, bcl-2 and caspase 3) and MAPK/NF-κB pathway-related molecules (erk, p-erk, p38, p-p38, p65 and p-p65) examined by qRT-PCR or Western blotting assays. (**G**) Each of the protein bands. The dividing lines (dashed lines) indicated that the grouping of images were from different parts of the same gel. (**H**–**J**) Invasion and migration rates were analyzed when treated with AG-126, SB203580, SP600125 and BAY-11-7082 inhibitors.

In this study, cells were strained with Annexin V/PI and subjected to flow cytometry to determine the apoptotic cells. The results indicated that silencing of THRSP could inhibit HCC cell apoptosis (Figure 8A, 8B). qRT-PCR and Western blotting assays were performed to detect the cell apoptosis-related molecules, including bax, bcl-2 and caspase 3. The results indicated that silencing of THRSP significantly reduced the expression of bax and caspase 3, while enhanced the expression of bcl-2 (Figure 8C, 8D).

### THRSP regulated HCC cell progression by modulating MAPK/NF-κB signaling pathway

To further understand the molecular mechanism by which si-THRSP promoted the migration and invasion of HCC cells, we explored the potential signaling pathways including the NF-κB and MAPK signaling pathways by Transwell and wound healing assays. As shown in Figure 8H–8J, compared with negative control, the migration and invasion of HCC cells transfected with si-THRSP or si-NC were inhibited after being treated with BAY-11-7082 (NF-κB inhibitor), AG-126 (ERK1/2 inhibitor), SB203580 (p38 MAPK inhibitor) and SP600125 (JNK inhibitor). And there was no difference between the si-THRSP group and the si-NC group when the cells were treated with BAY-11-7082, AG-126 and SB203580, indicating that the cell migration and invasion caused by the siRNA-induced silencing of THRSP might depend on the NF-κB, ERK1/2 and p38 MAPK signaling pathways. qRT-PCR and Western blotting assays were performed to assess the expression of MAPK/NF-κB pathway-related molecules (p65, p-p65, p38, p-p38, erk1/2 and p-erk1/2). The results showed that the silencing of THRSP increased the phosphorylation of ERK1/2, p38 MAPK and p65, and enhanced the expression of p65, p38, and erk1/2 (Figure 8E, 8F). Each of the protein bands were showed in Figure 8G.

## DISCUSSION

HCC is highly malignant with a poor prognosis [13]. Despite advances in radiotherapy, chemotherapy, and surgical resection over the past decades, the 5-year survival rate of HCC remains frustrating [14]. There is an urgent need to identify effective molecular targets to improve diagnostic and therapeutic approaches for HCC. Herein, integrated bioinformatic analysis was performed to identify effective molecular targets on two GEO datasets and the TCGA database. Six genes (namely LCAT, ACSM3, IGF1, SRD5A2, THRSP, and ACADS) were finally defined as survival-related hub genes. The roles of THRSP in HCC progression have rarely been reported. Here, we aimed to investigate biological functions and underlying mechanisms of THRSP in regulating HCC.

In this study, we systematically analyzed the mRNA expression, epigenetic modifications, immune significance, and clinical value of THRSP in HCC by bioinformatics analysis. The results indicated that the expression of THRSP was negatively correlated with its methylation and closely correlated with several clinical characteristics in HCC patients. The HCC patients with higher expression of THRSP have better 5-year survival. In addition, THRSP expression was negatively correlated with most of the immune cells, and it might play an important role in the tumor microenvironment of HCC. The further function experiments implicated that silencing of THRSP could promote cell proliferation, migration, invasion and cell division, and inhibited apoptosis of HCC cells. NF-κB, ERK1/ERK2, and p38 MAPK signaling pathways were vital for THRSP- mediated HCC progression. Eventually, we concluded that THRSP may be a promising therapeutic target for HCC.

For the other hub genes related to the prognosis of HCC, LCAT (Lecithin-cholesterol acyltransferase) is a plasma enzyme involved in reverse cholesterol transport (RCT) and high-density lipoprotein (HDL) metabolism and has been reported to play an important role in many other cancers, such as breast cancer [15], Hodgkin lymphoma [16], and ovarian cancer [17]. The previous studies had reported the significantly low expression and high DNA methylation of LCAT in HCC patients [18–20]. Besides, LCAT plays a crucial role in the conversion of liver cirrhosis into HCC [21]. ACSM3, as one member of the acyl-CoA synthetase medium-chain family, was found to be frequently down-regulated in HCC patients exhibiting high AFP levels, high ALT levels, large tumors, and multiple nodules. On the contrary, higher ACSM3 expression was always associated with a better prognosis and may hinder metastasis of HCC by downregulating phosphorylation of WNK1 and AKT [22]. IGF-1 (growth factor-1) has been widely reported that its expression decreased sharply in patients with chronic liver disease such as steatosis, nonalcoholic steatohepatitis, chronic hepatitis C, cirrhosis, and HCC [23–29]. The reason may be that most of the circulating levels of IGF-1 were synthesized by the liver [30, 31]. IGF1 synthesis decreases when hepatitis or liver necrosis occurs. In addition, a prospective cohort study demonstrated that IGF-1 can be an independent predictor of survival or recurrence in early HCC [32]. IGF1 was also demonstrated to play an important role in the cellular function aspects of hepatocarcinogenesis and could be a therapeutic target against HCC [33–35]. For instance, Sorafenib could inhibit macrophage-induced growth of hepatoma cells by disrupting IGF1 secretion [34]. SRD5A2, also known as steroid 5-alpha-reductase 2, encodes a microsomal protein. As a membrane-associated enzyme, it catalyzes the transformation of testosterone to dihydrotestosterone (DHT). SRD5A2 is highly expressed in androgen-sensitive tissues such as the prostate and the expression of SRD5A2 is associated with the progression of prostate cancer [36–39]. To date, some reports revealed that SRD5A2 polymorphism may be associated with liver cancer, and it might serve as a robust diagnosis or prognosis marker for the diagnosis of HCC [40–42]. ACADS, namely acyl-CoA dehydrogenase short-chain, encodes a tetrameric mitochondrial flavoprotein and catalyzes the initial step of the mitochondrial fatty acid beta-oxidation pathway. It was identified as a potential biomarker in colon adenocarcinoma and bladder cancer [43, 44]. A previous study demonstrated that ACADS was significantly down-regulated in HCC tissues and was regulated by DNA methylation, which played a key role in promoting the proliferation and metastasis of HCC [45].

In summary, by a series of comprehensive bioinformatics analyses, our study screened six significant survival-related hub genes. Among them, we found a novel biomarker (THRSP) associated with HCC development. The experimental results showed that lower expression of THRSP can promote the progression of HCC cells. Therefore, THRSP has the potential to be a valuable therapeutic target for HCC.

## MATERIALS AND METHODS

### Datasets preprocessing

Two gene expression profiles GSE84005 and GSE121248 were obtained from the GEO database. The GSE84005 dataset, including 38 tumor tissues and paired 38 normal tissues from HCC patients, was based on the GPL5175 platform (Affymetrix Human Exon 1.0 ST Array). The GSE121248 dataset, including 70 HCC tissue samples and 37 adjacent non-tumor tissue samples, was based on the GPL570 platform (Affymetrix Human Genome U133 Plus 2.0 Array). Two datasets were merged by Perl 5.3 (available online: http://www.perl.org/) to increase the sample size. The merged dataset was batch-normalized by “limma” and “sva” packages in R 3.6.3 (https://www.r-project.org/) to remove batch effects.

Besides, the RNA-sequencing (RNA-seq) data of 373 HCC and 49 normal samples and the corresponding clinical information was downloaded from the TCGA database. As recommended by the package “edgeR” in R, genes with low read counts (count per million (cpm) ≤ 1) were omitted. Gene expression was calculated and normalized to RPKM (reads per kilobase per million) values using function “rpkm” in the “edgeR” package. Moreover, DNA promoter methylation data (Methylation 450k, including 430 samples) was downloaded from the TCGA database via the UCSC Xena browser (https://xenabrowser.net/).

### WGCNA analysis

The “WGCNA” package in R was applied to construct co-expression networks and to explore the key modules of highly relevant genes by cluster analysis for relating modules to sample traits. In this study, the gene expression profiles of TCGA-LIHC and the merged dataset of GSE84005 and GSE121248 were respectively used to construct WGCNA. Briefly: an adjacency matrix was created by Pearson’s correlations between each of the gene pairs. Next, the adjacency matrix was utilized to erect a scale-free co-expression network based on the soft threshold power β which was selected using the pickSoftThreshold function [46]. Subsequently, the adjacency matrix was converted into a topological overlap matrix (TOM) as well as the corresponding dissimilarity (1-TOM). Then, module identification was conducted using the dynamic tree Cut approach by average linkage hierarchical clustering based on the TOM-based dissimilarity measure with the parameters of minModuleSize of 50, deepSplit value of 2, and mergeCutHeight of 0.25 for the genes dendrogram. Afterward, the correlation between module eigengenes (MEs) and the clinical trait information was calculated by the module-trait relationship analysis of WGCNA to identify the clinically significant modules in a co-expression network. Finally, modules with a high correlation coefficient were selected for further analysis.

### Identification of differentially co-expressed genes

The “limma” package in R was used to filter the DEGs between the HCC and normal samples in the TCGA-LIHC and the merged dataset of GSE84005 and GSE121248. The adjusted P-value (adj. P) < 0.05 and |log2foldchange (FC)| >1 was set as the criteria of DEGs. Then, the overlapping genes between DEG lists and co-expression genes from significant modules were screened out using the “VennDiagram” package in R.

### Identification of hub genes

The PPI network was constructed in the STRING (http://string-db.org) and visualized by the Cytoscape software (Cytoscape_v3.8.0, https://cytoscape.org/) [47]. The maximal clique centrality (MCC) analysis was performed to extract the candidate hub genes with the top20 MCC values in the PPI network using the cytoHubba plug-in [48]. Meanwhile, the Molecular Complex Detection (MCODE) [49] plug-in of Cytoscape was implemented to find significant PPI modules with degree cut-off ≥2, node score cut-off ≥0.2, K-core ≥2, and max. depth =100. Finally, the overlapping genes obtained from the MCC analysis and MCODE analysis were regarded as hub genes.

### The gene expression and prognostic analysis of the hub genes

The differential expression analysis of the hub genes between HCC and normal tissues was performed based on the GEPIA2 database. The 5-year overall survival (OS) analysis of these hub genes was performed using the “survival” and “survminer” packages in R [50].

### The correlation between gene expression and clinical features

According to the median value of gene expression or methylation, the HCC patients were divided into the low- or high- group. The chi-square test was utilized to investigate the correlation of gene expression as well as methylation with clinical characteristics. The correlation between DNA methylation and gene expression in HCC samples from the TCGA database was examined using the Spearman correlation coefficient and visualized by “ggplot2” and “ggpubr” packages in R.

### Immune infiltrate analysis

The online tool TIMER 2.0 (https://cistrome.shinyapps.io/timer/) was used for immune infiltrate analysis [51]. The abundance of six types of immune cells (including CD8+ T cells, CD4+ T cells, B cells, neutrophils, macrophages, and dendritic cells) were computed by TIMER algorithm. In addition, we exploited the correlation between gene expression and the gene markers of different kinds of immune cells. The immune gene markers of interest used in this study were referred to the previous studies [52–56].

### Cell culture

The human normal liver cell line (LO2) and the human hepatoma cell lines (HCCLM3 and HUH-7) were purchased from Procell Life Science and Technology Co., Ltd. (Wuhan, China). The cells were cultured in DMEM (Servicebio Technology Co., Ltd, Wuhan, China) supplemented with 10% Fetal Bovine Serum (FBS, G-CLONE, Beijing, China) and maintained in a incubator with 5% CO2-humidified atmosphere at 37° C.

### Total RNA extraction and qPCR

The quantitative real-time polymerase chain reaction (qRT-PCR) was employed to detect the expressions of THRSP. GAPDH was served as a reference gene. Total RNA was extracted from tissues and cells by the TRIzol reagent (G-CLONE, Beijing, China). The expression of THRSP was determined by the SweScript RT I First Strand cDNA Synthesis Kit with gDNA Remover and the SYBR Green qPCR Master Mix (High ROX) (Servicebio Technology Co., Wuhan, China) according to the manufacturer’s protocol. The qRT-PCR was performed on the StepOne Plus Real-Time PCR Systems. The primers used in this study were as follows: THRSP forward: 5’-CAGGTGCTAACCAAGCGTTAC-3’, THRSP reverse: 5’-CAGAAGGCTGGGGATCATCA-3’; GAPDH forward: 5’-GGACCTGACCTGCCGTCTAG-3’, GAPDH reverse: 5’-GTAGCCCAGGATGCCCTTGA-3’.

### IHC analysis

10 pairs of HCC tissues and paracancerous tissues were fixed in formalin, dehydrated, and embedded in paraffin. The paraffin sections were deparaffinized for antigen retrieval and treated with 3% hydrogen peroxide for blocking peroxidase activity, with 3% bovine serum albumin (BSA) for serum sealing. Afterward, the paraffin sections were incubated with primary THRSP antibody (Guangzhou Alexan Biotech Co., Ltd., China) overnight at 4° C, and then with HRP-conjugated secondary antibody for 50 min at room temperature. 3,3′-Diaminobenzidine (DAB) liquid substrate was used for staining and the hematoxylin solution was used for nucleus counterstaining. Finally, after dehydration and mounting, a microscope was used to acquire images of the staining of tissues.

### Transfection of small interfering RNA

Three short interfering RNAs (siRNAs) of THRSP (T1: 5’-ACACCTACTTCACCATGCT-3’; T2: 5’-CCAGGAAATGACGGGACAA-3’; T3: 5’-CATGCACCTCACCGAGAAA-3’) and negative control siRNA (si-NC) were purchased from RiboBio Co., Ltd. (Guangzhou, China). HCCLM3 and Huh-7 cells (2×10^5^ per well) were inoculated on 24-well plates for 24h and then transfected with 25pmol of the RNA duplex according to the manufacturer’s protocol of Lipofectamine 2000 (Invitrogen, Grand Island, NY, USA). After 24h, the transfected cells were harvested for the following experiments.

### CCK-8 assay

The transfected HCCLM3 and Huh-7 cells were seeded into 96-well plates (3×10^3^ cells/well) and incubated for 24h, 48h and 72h. At each time point, 10μl of CCK-8 reagent (Guangzhou Alexan Biotech Co., Ltd., China) was dripped into each well and the cells were cultured for an extra 4h. A micro-plate reader was used to detect the absorbance at 450nm to evaluate cell proliferation.

### Transwell and scratch wound healing assays

Transwell assays were used to determine the migration and invasion ability of the HCC cells. The transfected HCCLM3 cells and Huh-7 cells in 200μl serum-free DMEM (5×104 cells/well) were plated into the upper chamber and 600μl of complete medium was added in the lower chamber. For cell invasion assay, the Transwell chamber was coated with Matrigel (Beijing Solarbio Science and Technology Co., Ltd., China). After incubating at 37° C for 24h, the remaining cells in the upper chamber were removed by a cotton swab. The cells that have invaded to the lower surface of the filter were fixed with 4% (v/v) neutral formaldehyde solution (Servicebio, Wuhan, China) for 30min and stained with 0.1% Crystal violet for 30min. Finally, the cells in ten random microscope fields of each filter were counted.

The transfected HCCLM3 and Huh-7 cells (5×10^5^ cells per well) were seeded into 24-well plates. When the cell confluence reaches 90%, a scratch wound was created using a sterilized pipette tip (200μl) on confluent cells. The images of wounds were acquired with a phase-contrast light (40×) at 0h and 24h. The heal area of each scratch wound was determined by ImageJ.

To further understand the molecular mechanism, the potential signaling pathways including the NF-κB and MAPK signaling pathways were detected by Transwell and wound healing assays. Briefly: post of transfection for 48h, the cells were harvested and pre-treated with BAY-11-7082 (5μM, NF-κB inhibitor), AG-126 (10μM, ERK1/2 inhibitor), SB203580 (10μM, p38 MAPK inhibitor) and SP600125 (50μM, JNK inhibitor) for 2h in serum-free DMEM medium. Then, the cells were cultured in the DMEM medium containing inhibitors for 24 hours after being seeded into Transwell chambers or culture wells. The cells treated with dimethyl sulfoxide (DMSO) were used as the negative control.

### Flow cytometry

For the cell cycle assays, after 48h of transfection, the HCCLM3 cells were trypsinized, and washed with cold phosphate-buffered saline (PBS) and then fixed in 70% cold ethanol at 4° C for 24 h. After centrifuging and washing, the cells were stained with 500μl PI buffer (50μg/mL, containing RNase, Beijing Leagene Biotechnology Co., Ltd., China) at 37° C in the dark for 30 min. The cell cycle distribution was determined by the Flow cytometer after PI staining. The apoptosis analysis was performed following the instruction of Annexin-V Apoptosis Detection kit (Jiangsu KeyGEN BioTECH Co., Ltd., China). Briefly, 72h post-transfection, the trypsinized cells were washed with cold PBS twice and re-suspended in binding buffer, and then stained with Annexin V-FITC and PI at room temperature for 5-15 min in the dark. The apoptotic cells were analyzed by flow cytometry within an hour.

### Western blotting

The total protein was extracted with the nucleoprotein and cytoplasmic protein extraction kit (Jiangsu Keygen Biotech Co., Ltd. China) and quantified by the BCA protein assay kit (Beijing Bomaide Gene Technology Co., Ltd., China). After that, the protein solution was subjected to electrophoresis, detached via SDS-PAGE (12% gels), and transferred to PVDF membranes. The PVDF membranes were blocked with 5% skim milk powder in TBST (Tris buffered saline with 0.5% Tween 20) for 2 h, and then incubated with primary antibodies overnight at 4° C. After washing with TBST for 5 times, the PVDF membranes were incubated with secondary antibody at room temperature for an hour. The protein bands were exposed by enhanced chemiluminescent (ECL) substrate kit (Labgic Technology Co., Ltd. Hefei, China) and analyzed by ImageJ software. The Anti-Bcl-2 antibody, Anti-Caspase-3 antibody, Anti-Bax antibody, Anti-NF-κB p65 antibody and Anti-NF-κB p65 (phospho S536) antibody were purchased from Abcam. The p38 MAPK, Phospho-p38 MAPK (Thr180/Tyr182), P44/42 MAPK (Erk1/2), and Phospho-p44/42 MAPK (Erk1/2) (Thr202/Tyr204) were purchased from Cell Signaling Technology. β-actin was severed as the internal control and anti-β-actin was purchased from Labgic Technology Co., Ltd.

### Ethics approval

The manuscript has been approved by the ethics committee of the affiliated hospital of Southwest Medical University (Ethical ID: KY2021142).

## Supplementary Material

Supplementary Figures

Supplementary Table 1
