# Supplementary material for: THRSP identified as a potential hepatocellular carcinoma marker by integrated bioinformatics analysis and experimental validation
Source: Aging (Albany NY). 2022 Feb 23;14(4):1743–66. doi: 10.18632/aging.203900 (PMC8908915; doi:10.18632/aging.203900)
Supplement: Supplementary Figures [file aging-14-203900-s001.pdf]

SUPPLEMENTARY FIGURES

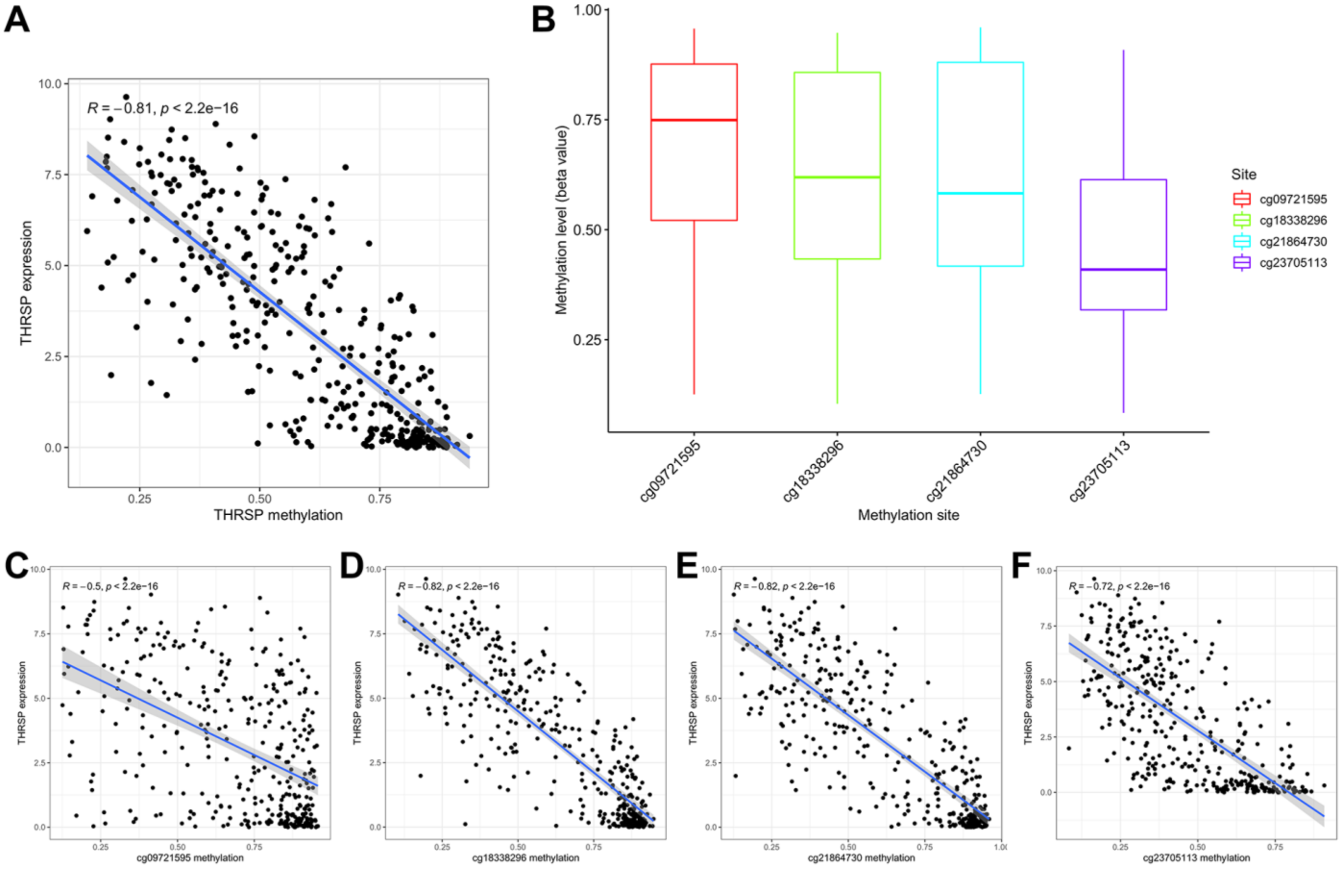

Supplementary Figure 1. Relationship between THRSP mRNA expression and DNA methylation. (**A**) THRSP expression was negatively correlated with DNA methylation (R = − 0.81, p < 2.2e-16). (**B**) The distribution of four THRSP DNA promoter CpG sites. (**C**–**F**) THRSP expression was negatively correlated with the methylation levels of the four CpG sites. From the left to right were cg09721595 (R = − 0.5, p < 2.2e-16), cg18338296 (R = − 0.82, p < 2.2e-16), cg21864730 (R = − 0.82, p < 2.2e-16) and cg23705113 (R = − 0.72, p < 2.2e-16).

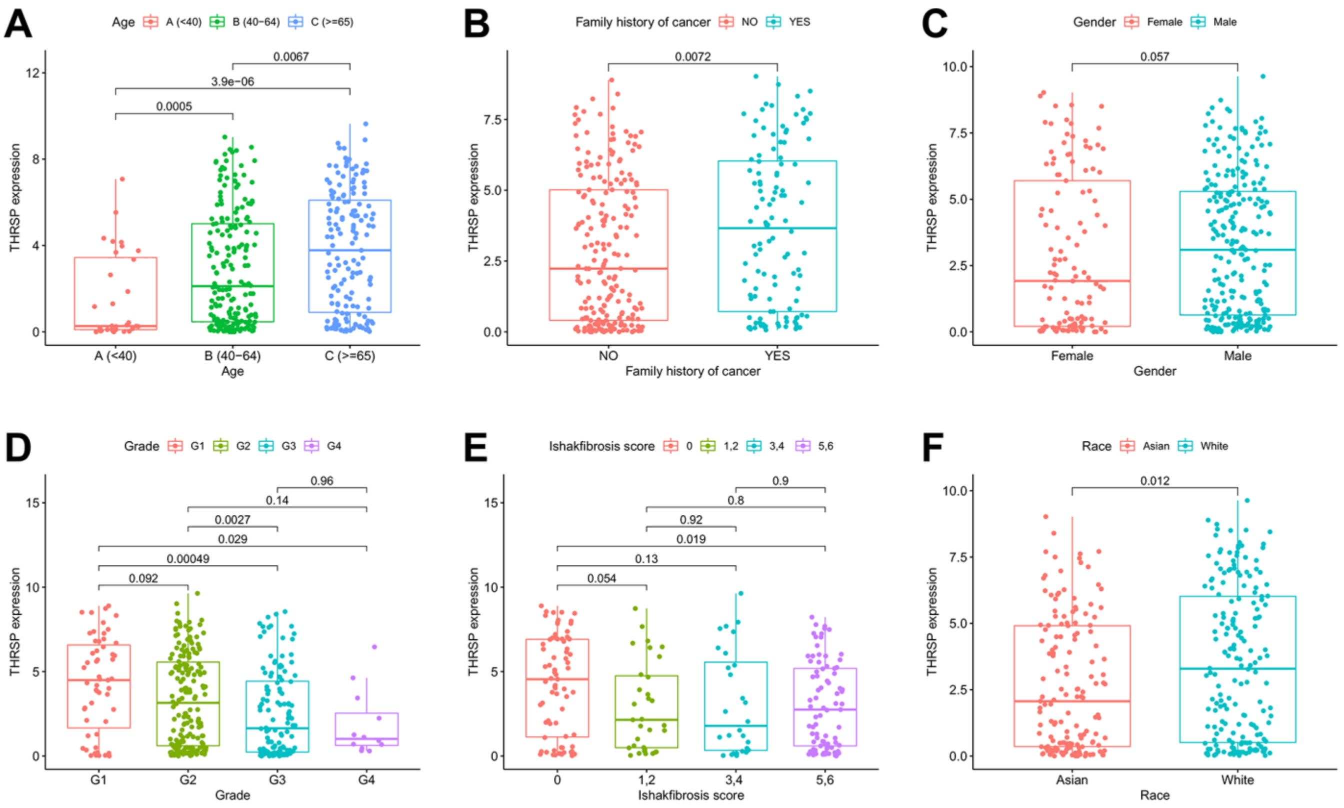

Supplementary Figure 2. The correlation between THRSP expression and the clinical features. THRSP mRNA expression was stratified by (**A**) age, (**B**) family history of cancer, (**C**) gender, (**D**) grade, (**E**) Ishak fibrosis score, and (**F**) race.

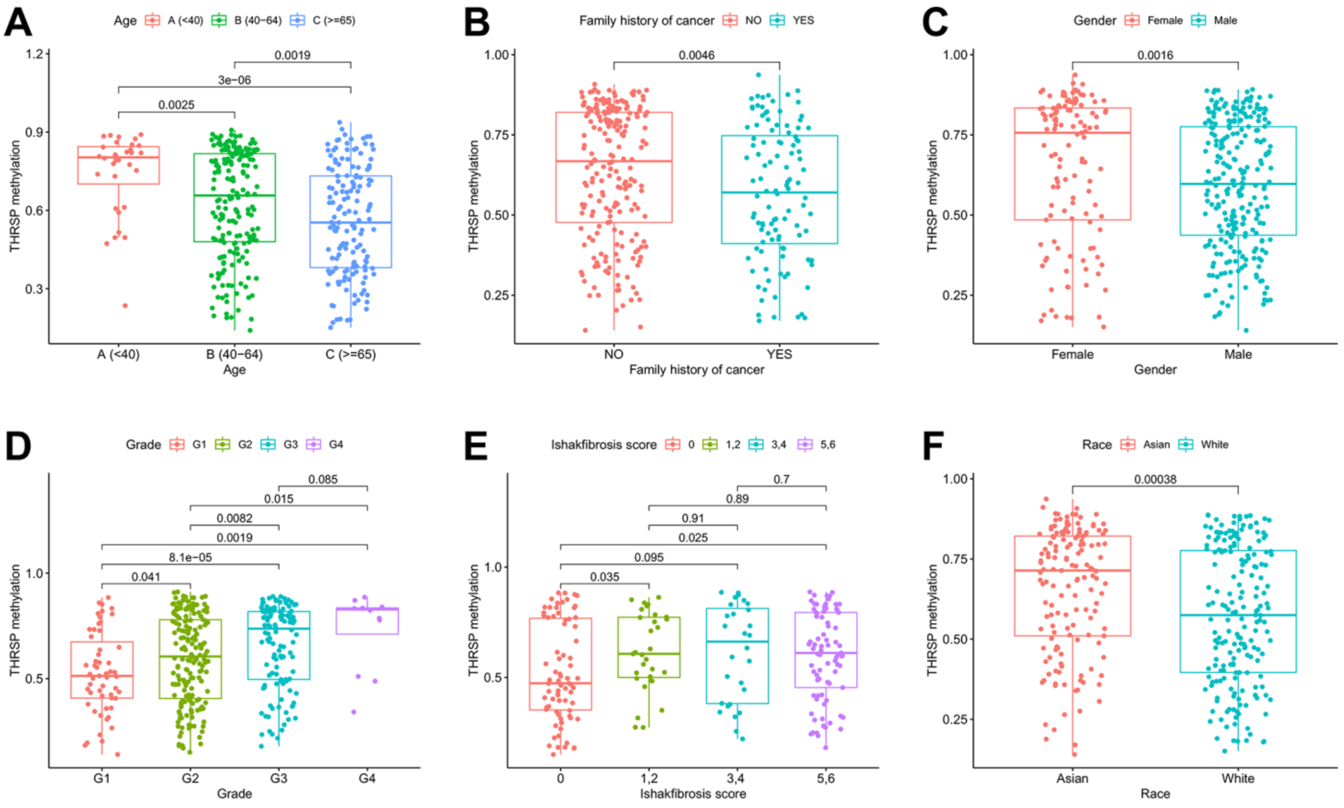

Supplementary Figure 3. The correlation between THRSP methylation and the clinical features. THRSP methylation was stratified by (**A**) age, (**B**) family history of cancer, (**C**) gender, (**D**) grade, (**E**) Ishak fibrosis score, and (**F**) race.

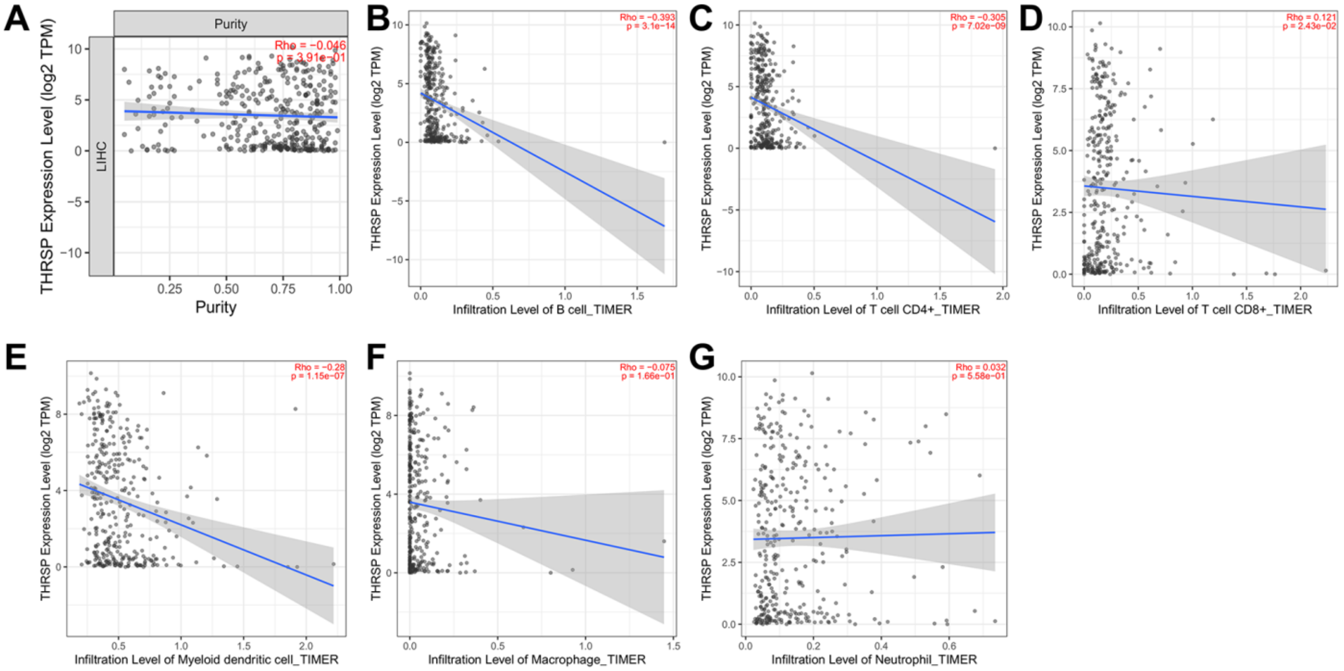

Supplementary Figure 4. The correlation between THRSP expression and immune infiltration levels in HCC. (**A**) No association between THRSP expression and tumor purity (R = −0.046, p = 0.391). (**B**–**G**) THRSP expression was significantly negatively correlated with infiltrating levels of B cells, CD4+ T cells, and dendritic cells, positively correlated with CD8+ T cells, but not correlated with macrophages or neutrophils.
