# Supplementary material for: THRSP identified as a potential hepatocellular carcinoma marker by integrated bioinformatics analysis and experimental validation
Source: Aging (Albany NY). 2022 Feb 23;14(4):1743–66. doi: 10.18632/aging.203900 (PMC8908915; doi:10.18632/aging.203900)
Supplement: Supplementary Table 1 [file aging-14-203900-s002.pdf]

SUPPLEMENTARY TABLE

Supplementary Table 1. Correlations between THRSP and related genes and markers of immune cells.

Description gene makers		LIHC			
		None		Purity	
		Cor	p	Cor	p
CD8+ T cell	CD8A	-0.067	0.198	-0.099	0.066
	CD8B	-0.078	0.134	-0.109	*
T cell (general)	CD3D	-0.152	**	-0.183	***
	CD3E	-0.105	*	-0.16	**
	CD2	-0.122	*	-0.176	**
	CD6	-0.111	*	-0.163	**
	CD3G	-0.108	*	-0.13	*
B cell	CD19	-0.118	*	-0.14	**
	KIAA0125	-0.126	*	-0.166	**
	SPIB	-0.242	***	-0.265	***
	PNOC	-0.161	**	-0.22	***
	CD79A	-0.132	*	-0.181	***
Monocyte	CD86	-0.143	**	-0.198	***
	CSF1R	-0.108	*	-0.167	**
Macrophages	CD68	-0.184	***	-0.218	***
	CD84	-0.004	0.946	-0.047	0.388
	MS4A4A	-0.034	0.512	-0.084	0.12
TAM	CCL2	0.039	0.452	0.033	0.541
	CD68	-0.184	***	-0.218	***
	IL10	-0.052	0.319	-0.093	0.086
	CSF1R	-0.108	*	-0.167	**
M1	IRF5	-0.061	0.241	-0.065	0.228
	NOS2	0.126	*	0.106	*
	PTGS2	-0.006	0.907	-0.028	0.605
M2	CD163	0.022	0.67	-0.012	0.831
	VSIG4	0.065	0.211	0.04	0.455
	MS4A4A	-0.034	0.512	-0.084	0.12
	MRC1	0.162	**	0.144	**
Neutrophils	CEACAM8	-0.06	0.246	-0.077	0.152
	ITGAM	0.052	0.322	0.03	0.578
	CCR7	-0.013	0.809	-0.049	0.362
	FCGR3B	0.154	**	0.154	**
	SIGLEC5	-0.11	*	-0.17	**
	CSF3R	-0.138	**	-0.19	***
Dendritic cell	HLA-DPB1	-0.151	**	-0.208	***
	HLA-DQB1	-0.13	*	-0.177	***
	HLA-DRA	-0.091	0.08	-0.139	**
	HLA-DPA1	-0.101	0.052	-0.157	**
	BDCA-1	-0.14	**	-0.17	**
	BDCA-4	-0.192	***	-0.194	***
	ITGAX	-0.111	*	-0.162	**
	HSD11B1	0.523	***	0.522	***
	CD209	-0.017	0.743	-0.04	0.46

	STAT4	0.002	0.97	0.002	0.974
	STAT1	-0.155	**	-0.167	**
Th1	TBX21	-0.012	0.82	-0.05	0.351
	IFNG	-0.047	0.362	-0.078	0.149
	TNF	-0.103	*	-0.151	**
	STAT5A	-0.126	*	-0.137	*
	IL13	0.287	***	0.297	***
Th2	GATA3	-0.079	0.127	-0.124	*
	STAT6	-0.099	0.056	-0.108	*
	CXCR4	-0.186	***	-0.222	***
Th1-like	BHLHE40	0.305	***	0.3	***
Th17	RORC	0.251	***	0.285	***
	CCR6	-0.313	***	-0.33	***
	CCR8	-0.06	0.251	-0.09	0.096
Treg	STAT5B	0.006	0.908	0.006	0.917
	TGFB1	-0.212	***	-0.246	***
Resting Treg T-cell	IL2RA	-0.139	**	-0.189	***
	FOXP3	0.211	***	0.198	***
Effective Treg T-cell	CTLA4	-0.224	***	-0.269	*
	TNFRSF9	-0.103	*	-0.129	*
Naïve T-cell	TCF7	-0.15	**	-0.17	**
Effective memory T-cell	DUSP4	-0.27	***	-0.328	***
	ITGAE	-0.443	***	-0.45	***
Resistant memory T-cell	CXCR6	-0.072	0.167	-0.119	*
	MYADM	-0.235	***	-0.243	***
	HAVCR2	-0.127	*	-0.185	***
	TIGIT	-0.11	*	-0.153	**
	LAYN	-0.129	*	-0.149	**
Exhausted T-cell	PDCD1	-0.209	***	-0.247	***
	CTLA4	-0.224	***	-0.269	***
	LAG3	-0.114	*	-0.121	*
	PTGER4	-0.124	*	-0.149	**

LIHC, Liver hepatocellular carcinoma. None, correlation without adjustment. Purity, correlation adjusted by purity.
P-value: 0 ≤ *** <0.001 ≤ ** < 0.01 ≤ * < 0.05.
